# Estimating the impact of birth interval on under-five mortality in east african countries: a propensity score matching analysis

**DOI:** 10.1186/s13690-023-01092-5

**Published:** 2023-04-21

**Authors:** Getayeneh Antehunegn Tesema, Misganaw Gebrie Worku, Tesfa Sewunet Alamneh, Achamyeleh Birhanu Teshale, Yigizie Yeshaw, Adugnaw Zeleke Alem, Hiwotie Getaneh Ayalew, Alemneh Mekuriaw Liyew, Zemenu Tadesse Tessema

**Affiliations:** 1 Department of Epidemiology and Biostatistics, Institute of Public Health, College of Medicine and Health Sciences and comprehensive specialized hospital, University of Gondar, Gondar, Ethiopia; 2 Department of Midwifery, School of Nursing and Midwifery, college of Medicine and health sciences, Wollo University, Dessie, Ethiopia; 3 Department of human anatomy, College of Medicine and Health Sciences and comprehensive specialized hospital, University of Gondar, Gondar, Ethiopia; 4 Department of human physiology, College of Medicine and Health Sciences and comprehensive specialized hospital, University of Gondar, Gondar, Ethiopia

**Keywords:** Propensity score matching, Under-five mortality, East Africa, Birth interval

## Abstract

**Background:**

Under-five mortality remains a global public health concern, particularly in East African countries. Short birth interval is highly associated with under-five mortality, and birth spacing has a significant effect on a child’s likelihood of survival. The association between short birth intervals and under-five mortality was demonstrated by numerous observational studies. However, the effect of short birth intervals on under-five mortality has not been investigated yet. Therefore, this study aimed to investigate the impact of short birth intervals on under-five mortality in East Africa using Propensity Matched Analysis.

**Methods:**

A secondary data analysis was conducted based on the most recent Demographic and Health Survey (DHS) data of 12 East African countries. A total weighted sample of 105,662 live births was considered for this study. A PSM analysis was carried out to evaluate the effect of short birth intervals on under-five mortality. Under-five mortality was the outcome variable, while the short birth interval was considered a treatment variable. To determine the Average Treatment Effect on the population (ATE), Average Treatment Effect on the treated (ATT), and Average Treatment Effect on the untreated (ATU), we performed PSM analysis with a logit-based model using the psmatch2 ate STATA function. The quality of matching was assessed statistically and graphically. The common support assumption was checked and fulfilled. We have employed Mantel-Haenszel bounds to examine whether the result would be free from hidden bias or not.

**Results:**

The prevalence of short birth intervals in East Africa was 44%. The under-five mortality rate among mothers who had optimal birth intervals was 39.9 (95% CI: 38.3, 41.5) per 1000 live births while it was 60.6 (95% CI: 58.5, 62.8) per 1000 live births among mothers who had a short birth intervals. Propensity score matching split births from mothers into treatment and control groups based on the preceding birth interval. In the PSM analysis, the ATT values in the treated and control groups were 6.09% and 3.97%, respectively, showed under-five mortality among births to mothers with short birth intervals was 2.17% higher than births to mothers who had an optimal birth interval. The ATU values in the intervention and control groups were 3.90% and 6.06%, respectively, indicating that for births from women who had an optimal birth interval, the chance of dying within five years would increase by 2.17% if they were born to mother with short birth interval. The final ATE estimate was 2.14% among the population. After matching, there was no significant difference in baseline characteristics between the treated and control groups (p-value > 0.05), which indicates the quality of matching was good.

**Conclusions:**

We conclude that enhancing mothers to have optimal birth spacing is likely to be an effective approach to reducing the incidence of under-five mortality. Our findings suggest that births to mothers with short birth intervals have an increased risk of death in the first five years of life than births to mothers who had an optimal birth interval. Therefore, public health programs should enhance interventions targeting improving birth spacing to reduce the incidence of under-five mortality in low-and middle-income countries like East African countries. Moreover, to achieve a significant reduction in the under-five mortality rate, interventions that encourage birth spacing should be considered. This will improve child survival and help in attaining Sustainable Development Goal targets in East African countries.

## Background

Under-five mortality is the probability of dying before five years of age, which is the key global indicator of child health [1]. Despite decades of success in reducing under-five mortality, neonatal, infant, and child mortality remain unacceptably high in many low- and middle-income countries [2, 3]. An estimated 6.3 million children under five died each year worldwide, with 2.9 million of those deaths occurring in the World Health Organization’s (WHO) African region [4, 5]. The global mortality estimates showed that the sub-Saharan Africa (SSA) region had the highest under-five mortality [6]. The majority of the deaths of under-five children are preventable [7].

The World Health Organization (WHO) currently recommends an interval between the last live birth and the next pregnancy of at least 24 months, a birth interval of 33 months [8]. Birth interval length, or the time between two consecutive live births, has been identified by the WHO as a significant predictor of the risk of under-five mortality, and it is advised that women space their pregnancies by three to five years to reduce the health risks to both mothers and children [9, 10]. The recommendation is based on research showing that intervals between 36 months and 60 months are linked to a higher risk of infant mortality and other negative effects. Women in countries with low or middle incomes are more likely to have a short birth interval [8].

Child survival is significantly influenced by birth spacing, and under-five mortality is substantially correlated with birth spacing that is not optimal [11]. Numerous studies have shown that short birth spacing is significantly linked with an increased risk of child mortality, preterm birth, small for gestational age, and low birth weight [12, 13]. These associations stem from the biological factor commonly referred to as maternal depletion syndrome [14]. Pregnancies that are closely spaced apart may not give the mother enough time to replenish her depleted micronutrient and macronutrient stores from the previous birthing, which could affect her ability to provide a favorable environment for fetal growth in subsequent pregnancies and sufficient breast milk production after delivery [15].

There is limited empirical evidence on the causal impact of preceding birth intervals on perinatal, infant, and child mortality. The adverse consequences of a short interval for infant and child survival and maternal mortality and morbidity have been attributed to the biological effects related to the “maternal depletion syndrome” or more generally the woman not fully recuperating from one pregnancy before supporting the next one [16, 17].

As advocated by the WHO, optimal birth spacing is one of the most effective strategies to reduce child mortality in low-and middle-income countries [18]. Extensive research has sought to understand the various factors associated with under-five mortality in East Africa. Numerous studies evidenced the association between birth interval and under-five mortality [19, 20]. However, the mothers may differ across known factors that influence birth interval and under-five mortality, i.e., they may be residence, education, wealth status, or access to health information. Therefore, traditionally to control for such confounding associations between short birth intervals and under-five mortality in statistical analysis has been done via regression analysis. However, since the regression analysis merely performs adjustments for the observable variables, bias (residual confounding) still exists. For instance, even when such factors are controlled for within the regression model, the variation in the distribution of factors influencing birth intervals among mothers who had short birth intervals and mothers who had optimal birth spacing could bias the effects of short birth intervals on under-five mortality. In addition, due to unobserved variables that could create bias, women with short birth intervals are more susceptible to under-five mortality than women with optimal birth intervals.

Propensity Score Matching (PSM) is a methodological technique that aimed to remove bias by matching treated (Short Birth Interval (SBI)) and un-treated (Optimal Birth Interval (SBI)) pregnant mothers with similar conditional probability to receiving the treatment (SBI). In this study, we matched the mothers with OBI to mothers with SBI with similar propensity score values for birth intervals [21, 22]. Then, it can be reasoned that any difference in under-five mortality is attributed to birth interval only. The use of propensity score matching is the appropriate approach to assess the impact of birth interval on under-five mortality compared to the standard regression adjustment. According to our literature search, there was no study conducted in East Africa that employed propensity score matching analysis to evaluate the effect of short birth intervals on under-five mortality. The present study addresses the methodical limitations of previous studies by examining the effect of short birth intervals on under-five mortality among mothers in East Africa using PSM analysis.

## Methods

### Study design and period

A secondary data analysis was done based on the most recent Demographic and Health Survey (DHS) data of the 12 East African countries conducted from 2012 to 2021. DHS is a community-based cross-sectional study conducted in a five-year interval to generate updated health and health-related indicators.

### Sample size and sampling techniques

The DHS survey of 12 East African countries (Burundi, 10,626; Ethiopia, 8455; Kenya, 16,106; Comoros, 2413; Madagascar, 9499, Malawi, 12,782; Mozambique, 8542; Rwanda, 5495, Tanzania, 7805; Uganda, 12,146; Zambia; 7424; and Zimbabwe, 4428) was used for the current study. The total sample size at the East African level was 105,721. Only mothers who had a previous history of birth were included in this study.

### Outcome and treatment variables

The outcome variable was under-five mortality. It was coded as “0” if the child was alive and “1” if the child died within the first 59 months of age. Pregnant mothers who had a previous history of birth were considered for this study (primigravida mothers were excluded). The treatment group is those mothers who had a short birth interval and the control group is those who had an optimal birth interval. According to the WHO recommendation, the birth interval was divided into two categories: “short birth interval,“ defined as less than 33 months, and “optimal birth interval,“ defined as 33 months and above. Many maternal pre-intervention characteristics have been included in the model as it ensures a better chance that the propensity score matching assumption holds. Variables that affect short birth intervals and under-five mortality but which are not affected by the treatment (SBI) were included. Variables such as residence, country, and mother’s level of education, household wealth status, sex of household head, marital status, number of birth before the current birth, maternal age, and media exposure were considered for matching.

### Data management and analysis

In randomized control trials, study participants are assigned to either the treatment or control group through randomization that controls both known and unknown confounders between the groups (no systematic difference between groups). whereas, because researchers cannot randomly assign study participants to either of the groups in observational studies, the imbalance of the observed variables introduces bias and affects the exposure’s causal effect. In situations where confounding variables can be measured, we can adjust for and treat the imbalance between groups. A function of the observed covariates so-called the balancing score can be used to correct the imbalance between the groups. Based on the balancing score, the observed variables should be independent of the assignment of the treatment i.e. short birth interval or optimal birth spacing.

The propensity score method is commonly used to balance the inequality of confounding variables in observational studies. After adjusting for the observed covariates using propensity scores, the difference in outcomes between the child born to mothers with short birth intervals and the child born to mothers with optimal birth intervals will be an unbiased estimate of the effect of short birth intervals on under-five mortality. A propensity score is the likelihood that a pregnant woman received a treatment (short birth interval) given all the observed covariates. It is a conditional probability of receiving treatment (SBI) and thus always has a value between 0 and 1. The larger the propensity score, the more likely a woman is to have SBI. The treatment variable of interest must be dichotomous in a propensity score analysis.

Propensity score analysis usually starts with an assessment of the imbalance of the baseline covariates between the treatment and control groups. This can be assessed by significance tests like an independent t-test for continuous variables and chi-square for categorical variables.

In the propensity score model, the exposure variable is considered a treatment variable, and the dependent variable is considered an outcome variable. Based on the relationship between treatment and outcome variables, observed covariates can be categorized into three groups: covariates only related to treatment assignment; covariates related to both treatment assignment and outcome (i.e., confounders); and covariates only related to outcome. However, PSM only can be included. The likelihood of the woman having SBI based on the selected confounders is reduced to a propensity score for each woman. This propensity score is generated for each subject from the selected confounders. Since the treatment variable of interest is dichotomous, the common methods adopted to produce propensity scores are either logistic regression or discriminant analysis.

Propensity score matching was used to assess the impact of short birth intervals on under-five mortality. The PSM is a statistical technique that aims to address the primary drawback of causal inference in observational research designs when randomization cannot be used to establish the treatment and control groups. This approach involves forming matching sets of control and treatments of individuals whose propensity scores are similar. After a matching sample has been established, the effect of SBI can be assessed by comparing under-five mortality directly between SBI and OBI women in the matched sample.

The PSM approach was employed to compare under-five mortality among births with optimal birth intervals and births with short birth intervals. The PSM approach was chosen because the birth interval was not randomly assigned to both groups and can be affected strongly by observable and non-observable variables. Variables such as socio-demographic and obstetrical-related variables, which have a significant association with birth interval and under-five mortality were selected as PSM variables. Births with short birth intervals were matched to births with optimal birth intervals using logit regression (***teffects psmatch STATA command***). Besides, we used a t-test to access the balance for all covariates before and after matching, with a 5% level of significance or more considered indicative of imbalance.

Before PSM, the baseline variables (i.e. country, residence, household wealth status, mothers level of education, sex of household head, parity, maternal age, marital status, and media exposure) showed significant differences (p < 0.05) in under-five mortality between the SBI and OBI groups. After matching, the abovementioned variables showed a non-significant difference (p-value > 0.05) in under-five mortality between mothers with SBI and mothers with OBI, suggesting that PSM significantly reduced the between-group difference in the observed characteristics.

We aim to estimate the average effect of short birth intervals on the treated. Assume A*iT* be under-five mortality for those i^th^ birth with short birth interval (treatment group), and A*iC* denotes under-five mortality for mothers with optimal birth intervals. The observed outcome can be written as $Ai=\left(1-Ti\right)AiC+TiAiJ$, where Ti = 0, 1 denotes treatment assignment (birth interval). The gain from the treatment (SBI) is ( A*i*^T^- A*i*^C^ ) and our interest is to estimate the average effect of treatment (SBI) on the treated (ATT), $E({Ai}^{T}-{Ai}^{C}/ Ti=1)$ [23]. This cannot be estimated directly since neither are normally observed as ${Ai}^{T}$ for Ti=0 and ${Ai}^{C}$ for Ti=1 are not known.

Overall variables for matching were selected before the treatment (short birth interval). The commonest assumptions of PSM such as common support and selection on unobservable were assessed graphically and statistically. During analysis, the common support option was considered to limit the balancing propensity to only mothers with treatment (SBI) whose propensity score for under-five mortality lay within ranges of propensity scores for controls. Using the ***pstest*** command to assess the covariate balance, we tested the following matching methods: nearest neighbor matching with and without replacement, and radius matching with calipers from 0.01. The ***psmatch2*** command was used to generate the ATT, ATU, and ATE for the matching method that produced the highest quality of matches. The common support option was also employed to produce higher-quality matches.

The quality of matching was evaluated based on the balancing of the covariates between the treated and control groups. Firstly, the quality of matching was assessed by computing the standardized bias before matching and after matching. This bias is computed as the percentage difference.

The bias is computed as the percentage difference of the sample means in the treated and matched control groups as a percentage of the square root of the average of the sample variances in both groups. Though no hard and fast rule exists on the level of standardized difference that would indicate an imbalance, a difference of less than 10% is taken to indicate a negligible difference. Secondly, the pseudo R^2^ and Likelihood ratio test of the joint insignificance of all the covariates from the logit estimation of the conditional treatment probability before and after matching.

A sensitivity analysis was conducted to test the robustness of the PSM estimates [24]. As the outcome variable was binary, the Mantel-Haenzel (MH) test statistic was used to check whether the PSM estimates are sensitive to the hidden bias [25]. The gamma coefficient is the factor by which the unobserved confounder or hidden bias affects the assignment of the intervention to the treated and control group. The gamma value ranges from 1 to 2 with a 0.05 increment using the **mhbounds** STATA command [26].

### Ethical considerations

Since the study was a secondary data analysis of publically available survey data from the MEASURE DHS program, ethical approval, and participant consent were not necessary for this particular study. We requested DHS Program, and permission was granted to download and use the data for this study from http://www.dhsprogram.com. There are no names of individuals or household addresses in the data files.

## Results

### Descriptive characteristics of the study participants

A total of 105,721 pregnant mothers who had a previous history of birth in 12 East African countries were included in this study. Of them, 46,489 (44%) of the mothers had a short birth interval. Under-five mortality among mothers who had optimal birth interval was 39.9 (95% CI: 38.3, 41.5) per 1000 live births whereas it was 60.6 (95% CI: 58.5, 62.8) per 1000 live births among mothers who had a short birth interval. All the baseline characteristics showed significant differences (p < 0.05) across the birth interval category (OBI vs. SBI) before matching (Table 1).

**Table 1:** The distribution of socio-demographic and obstetric related characteristics of the pregnant mothers with SBI and OBI, before propensity score matching

Variables	Before matching				
	NBI	Pct.	SBI	Pct.	p-value
**Residence**					
Urban	14,401	63.33	8340	36.67	< 0.001
Rural	44,831	54.03	38,149	45.97	
**Country**					
Burundi, 2016-17	5558	52.31	5068	47.69	< 0.01
Ethiopia, 2016	4268	50.48	4187	49.52	
Kenya, 2014	8767	54.43	7339	45.57	
Comoros, 2012	1126	46.66	1287	53.34	
Madagascar, 2021	4739	49.89	4760	50.11	
Malawi, 2015-16	9016	70.54	3766	29.46	
Mozambique, 2015	4894	57.29	3648	42.71	
Rwanda, 2019-20	3438	62.57	2057	37.43	
Tanzania, 2015-16	4054	51.94	3751	48.06	
Uganda, 2016	5622	46.29	6524	53.71	
Zambia, 2018	4611	62.11	2813	37.89	
Zimbabwe, 2015	3139	70.89	1289	29.11	
**Mothers educational status**					
No education	15,351	50.37	15,125	49.63	< 0.001
Primary	31,448	56.34	24,367	43.66	
Secondary	10,738	63.53	6164	36.47	
Higher	1695	67.05	833	32.95	
**Household wealth status**					
Poorest	14,443	48.71	15,205	51.29	< 0.001
Poorer	11,918	53.44	10,385	46.56	
Middle	11,228	57.19	8406	42.81	
Richer	10,933	60.36	7181	39.64	
Richest	10,710	66.85	5312	33.15	
**Sex of household head**					
Male	44,022	54.35	36,969	45.65	0.001
Female	15,210	61.50	9520	38.50	
**Marital status**					
Not married	16,023	58.22	11,497	41.78	0.01
Married	43,209	55.25	34,992	44.75	
**Number of births before the current birth**					
1–3	27,933	57.22	20,880	42.78	0.001
> 3	31,299	55.00	25,609	45.00	
**Maternal age (in years)**					
15–24	7066	40.14	10,537	59.86	< 0.001
25–34	32,682	56.73	24,927	43.27	
≥ 35	19,484	63.86	11,025	36.14	
**Media exposure**					
No	21,296	53.13	18,788	46.87	0.001
Yes	37,899	57.79	27,679	42.21	

### Estimations of propensity score

The strength of the association, the direction of the association, and the significance of the estimates were in line with previous researcher findings (Table 2). The mean propensity score was 0.44, with minimal variability (SD = 0.14) between the intervention and control groups. The common support assumption was satisfied and the region of common support was ranging from 0.0826 to 0.842 of the propensity score. Births from women in both intervention and control groups with propensity scores outside the region of common support were dropped.

**Table 2:** The association between covariates and short birth interval

Variables	Short birth interval	
	Coef. With 95% CI	P-value
Birth order	0.47 (0.43, 0.49)	< 0.001
Media exposure	0.03 (-0.003, 0.05)	0.082
Marital status	0.08 (0.05, 0.11)	< 0.001
Residence	0.06 (0.03, 0.09)	< 0.001
Sex of household	-0.25 (-0.28,-0.21)	< 0.001
Household wealth status	-0.12 (-0.13, -0.11)	< 0.001
Maternal educational status	-0.13 (-0.15, -0.11)	< 0.001
Maternal education	-0.66 (0.68, 0.64)	< 0.001
Constant term	0.80 (0.70, 0.90)	< 0.001
Number of observations	105,662	
LR chi2(8)	5567.88	
Prob > chi2	< 0.0001	
Pseudo R^2^	0.0384	
Log likelihood	-69686.91	

### Impact of birth interval on under-five mortality

The unmatched estimate showed that births from women who had short birth intervals had a 2.07% increased risk of under-five mortality compared to those born to women who had an optimal birth interval. A radius matching with a caliper width of 0.01 had the best quality of matching. The estimated average treatment on the treated values in the treated and control groups were 6.09% and 3.97%, respectively showing that the under-five mortality was increased by 2.12% because of the short birth interval. Likewise, the estimated average treatment effect on untreated values in the treated and control groups were 3.09% and 6.06%, respectively. This showed that if the babies born to optimal birth intervals were born to women with short birth intervals, the chance of dying in the first five years of life would have increased by 2.17%. Finally, the average effect on the whole population was found to be 2.14% among the total study population (Table 3).

**Table 3:** The impacts of short birth interval on under-five mortality in East Africa using PSM method

Impact of short birth interval on under-five mortality	Treated	Control	Difference	Standard error	t-statistic	p-value
Unmatched	0.0606	0.0399	0.0207	0.0013	15.53	
ATT	0.0609	0.0397	0.0212	0.0014	14.51	< 0.05
ATU	0.039	0.0606	0.0217			
ATE			0.0214			

*ATT: Average Treatment Effect among Treated, ATU: Average Treatment effect on Untreated, ATE: Average Treatment effect on the whole population

### Quality of matching

#### Common support

**Fig. 1:**
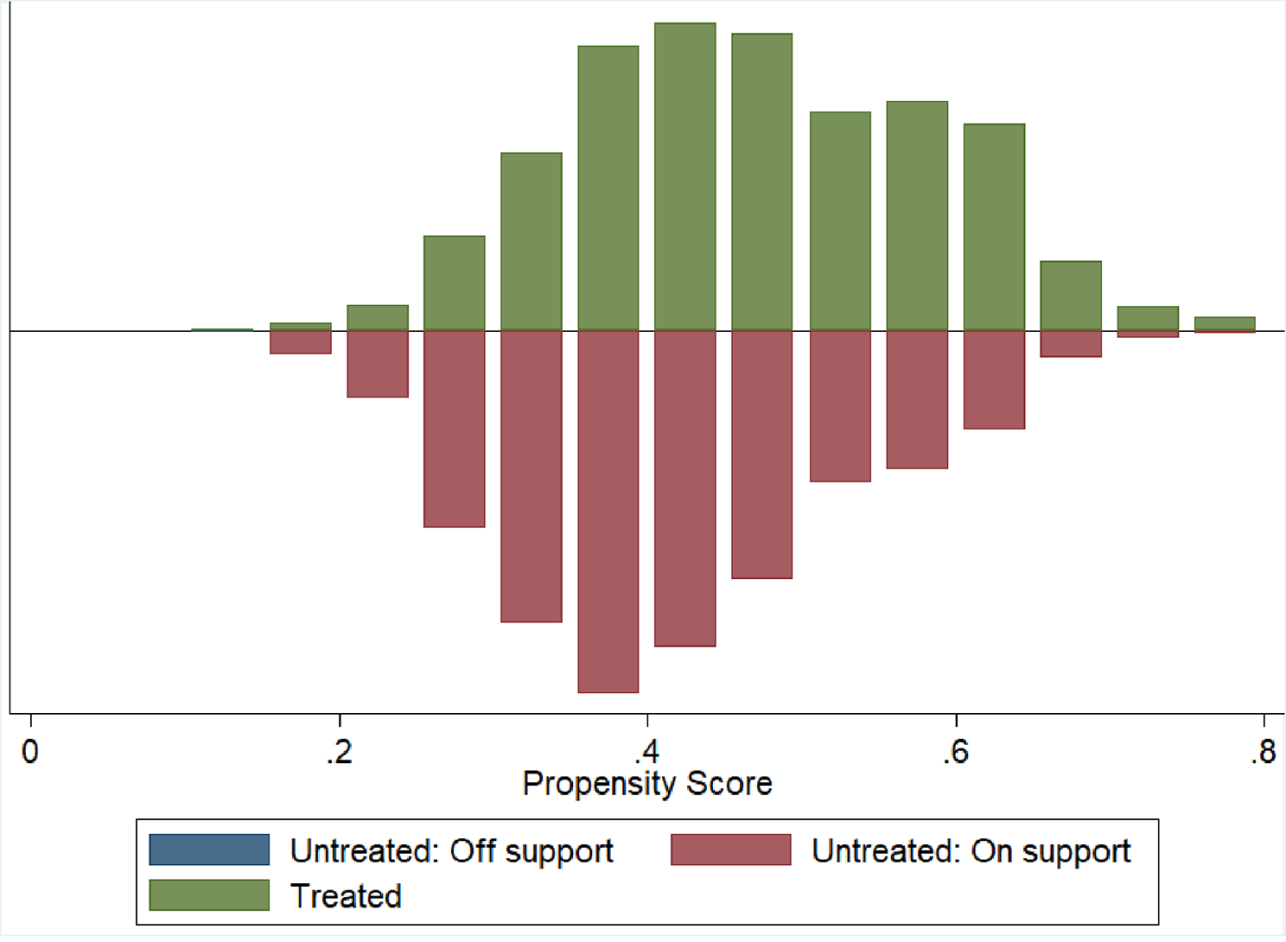
Propensity score histogram by treatment status (Short birth interval)

**Fig. 2:**
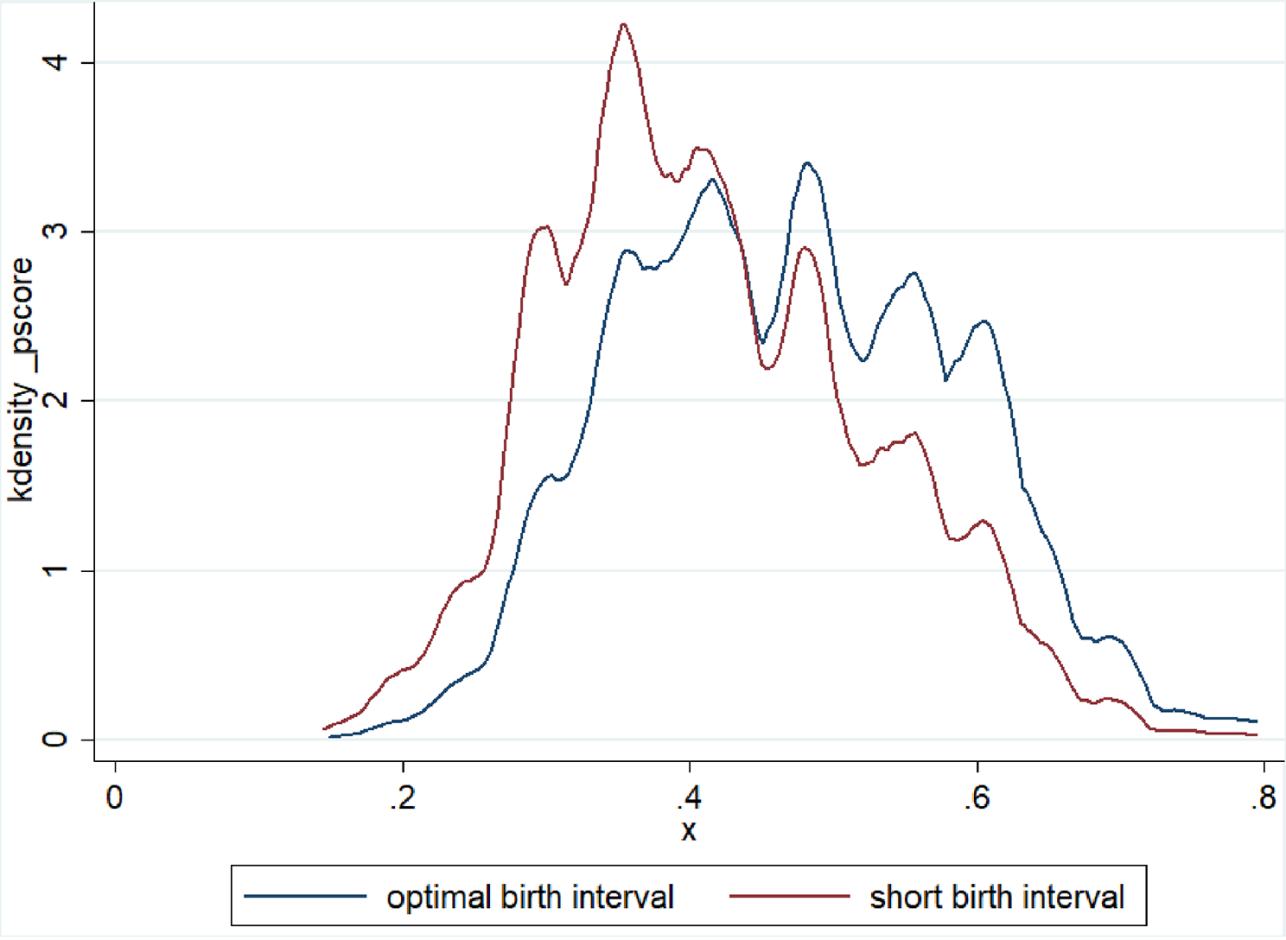
Propensity score distribution of short birth interval after matching

Only 2 women were discarded due to off-support (Table 4). We plot the distributions of propensity score and the distribution is almost similar for both the group’s post matching on PS (Figs. 1 and 2). The presence of significant overlap between the characteristics of the treated and control groups proves the validity of the common support assumption.

**Table 4:** Common support

Assigned treatment	Off support	On support	Total
Optimal birth interval	2	59,193	59,195
Short birth interval	0	46,467	46,467
Total	2	105,660	105,662

*The regions of common support was [0.0826, 0.842]*

### Balancing test

The difference between the unmatched and matched pairs was evaluated by t-test and the significance level of the test was reported. Though was a significant mean difference across all the covariates, there was no significant mean difference across almost all the covariates after matching (Table 5). This showed that the treated and control group are sufficiently balanced for all the variables included in the model.

**Table 5:** Performance of the propensity score matching: quality measurements

Variable	Sample	Mean		% bias	% Bias reduction	t-statistic	p-value
		Treated	Control				
Residence	Unmatched	0.82	0.76	15.7		25.12	< 0.001
	Matched	0.82	0.82	-0.2	98.6	-0.35	0.726
Media exposure	Unmatched	0.60	0.64	-9.2		-14.84	< 0.001
	Matched	0.60	0.60	-0.0	99.8	-0.03	0.979
Marital status	Unmatched	0.75	0.73	5.3		8.51	< 0.001
	Matched	0.75	0.75	0.0	99.3	0.06	0.952
Sex of household head	Unmatched	0.20	0.26	-12.4		-19.87	< 0.001
	Matched	0.20	0.20	0.1	99.2	0.15	0.877
Birth order	Unmatched	0.55	0.53	4.5		7.31	< 0.001
	Matched	0.55	0.55	0.0	99.3	0.05	0.963
Household wealth status	Unmatched	2.50	2.86	-25.0		-40.23	< 0.001
	Matched	2.50	2.50	0	99.8	0.07	0.947
Maternal education status	Unmatched	0.84	0.98	-18.7		-30.13	< 0.001
	Matched	0.84	0.84	0.2	99.1	0.26	0.796
Maternal age	Unmatched	1.01	1.21	-30.2		-48.97	< 0.001
	Matched	1.01	1.01	-0.0	99.9	-0.05	0.962

Matching approach = Radius matching with caliper 0.01

Mean bias Unmatched = 15.1: Matched = 0.1

Median bias Unmatched = 14.0: Matched = 0

Pseudo R^2^ Unmatched = 0.038: Matched < 0.001

LR chi-square Unmatched = 5564.79: Matched = 0.25

p-value Unmatched < 0.001: Matched = 1.00

### Standardized bias

In the pstest, there was a significant reduction in the mean and median biases after matching between the intervention and control groups. The mean absolute bias was decreased from 15.1% in the unmatched sample to 0.1% after matching between the treated and control group, which is less than the threshold (5%) showing the improvement of quality matching in the model. The median bias reduced from 14% in the unmatched to zero after matching (Table 5).

### Significance of the model

The overall significance of the model was assessed by the pseudo R^2^ and LR tests. The pseudo-R^2^ was < 0.001 and the LR-test had become insignificant (p = 0.25), showing that there was no systematic difference in covariate distribution between the treated and control groups (Table 5).

### Sensitivity analysis

The Mantel-Haenszel result showed that the overestimation of the effect of short birth intervals on under-five mortality was not significant at 5% of the level of significance. However, the underestimation of the effect of the short birth interval was found statistically significant at a 5% level of significance, the probability of underestimation of the effect of short birth interval on under-five mortality increases as the value of gamma increases (meaning that the odds of heterogeneity due to unobserved factors decreases) (Table 6).

**Table 6:** Sensitivity analysis using Mantel-Haenszel bounds

Gamma (Γ)	Test statistics		Significance level	
	Over-estimation (Q_mh+)	Under-estimation (Q_mh-)	Over-estimation (p_mh+)	Under-estimation (p_mh-)
1	1.40	1.40	0.08	0.08
1.05	1.17	1.63	0.12	0.05
1.1	0.95	1.85	0.17	0.03
1.15	0.74	2.06	0.23	0.02
1.2	0.55	2.26	0.29	0.01
1.25	0.36	2.46	0.36	0.007
1.3	0.17	2.65	0.43	0.004
1.35	-0.003	2.83	0.50	0.002
1.4	-0.042	3.01	0.52	0.001
1.45	0.12	3.18	0.45	0.0007
1.5	0.28	3.34	0.39	0.0004
1.55	0.43	3.50	0.33	0.0002
1.6	0.58	3.66	0.28	0.0001
1.65	0.72	3.82	0.24	0.00006
1.7	0.86	3.96	0.19	0.0003
1.75	0.99	4.11	0.15	0.00002
1.8	1.13	4.25	0.13	0.00001
1.85	1.25	4.39	0.104	0.00005
1.9	1.38	4.53	0.08	0.000003
1.95	1.50	4.66	0.07	0.000001
2.0	1.62	4.79	0.052	0.000008

## Discussion

In public health, healthcare decision-makers attempted to assess how the public health interventions among the treated populations have changed. This study investigated the impact of short birth intervals on under-five mortality using propensity score matching analysis. This approach is one of the best approaches to assess the impact of a certain intervention in observational studies by constructing an adequate comparison group since randomization can’t be applied.

Previous studies found that short birth interval was a significant predictor of under-five mortality and they have reported a statistically significant association between birth interval and under-five mortality in East African countries. However, we tried to estimate the impact of short birth intervals on under-five mortality and overcome the concern of selection on the unobservable. Women with short interbirth intervals were socio-economically, demographically, and birth-related characteristics are different at baseline than women who had an optimal birth interval. Therefore, conducting a standard regression analysis of under-five mortality between women with SBI and women with OBI would yield biased estimates of the impact of short birth interval on under-five mortality and the result would not be adjusted for endogeneity bias.

As to our review of literature, we found that existing literature showed that no study practically attempts to remove the selection bias/adjust at baseline to rectify the causal inference of short birth interval which leads to under-five mortality. Therefore, this study is the first study that attempts to quantify the effects of short birth intervals on under-five mortality using propensity scores after removing the possible selection bias.

Using PSM we found that births to mothers with short birth intervals had a 2.12% higher risk of death in the first five years of life compared to those births to women who had an optimal birth interval. Furthermore, for those births born to mothers who had an optimal birth interval, if they would have been born to mothers with short interbirth intervals, their chances of mortality in the first five years would have been increased to 2.17%.

The effects of short interbirth intervals on under-five mortality can be attributed to the fact that short birth intervals are associated with increased preterm birth, low birth weight, and small gestational age births, which in turn contribute to under-five mortality. The WHO recommends a healthy pregnancy gap of at least two years (24 months) [19], which equates to a birth-to-birth interval of 33 months on the assumption of a nine-month pregnancy. Under-five mortality is high for pregnancy intervals of fewer than 18 months [8]. Many African countries promote birth spacing as a key strategy to reduce the incidence of under-five mortality by making family planning services reachable [27–29]. Short birth interval has established a positive relationship with under-five mortality because the strong link between intervals to mortality because of sibling competition, which indicates closely spaced children are more likely to compete for the same resources such as breast milk, would be especially related to the length of a birth interval been reported to occur in the situation of shorter inter-birth intervals [10]. Besides, a family may invest more of its limited resources in the care of the newborn, and disease transmission could be common when the interbirth interval is too short [30]. Moreover, short interbirth intervals prevent a mother from recuperating in their nutrition and physical status, which is responsible for poor fetal development like low birth weight, IUGR, and preterm birth, which increases the risk of mortality during childhood [9, 17]. It is because micronutrients and muscle mass are being depleted from the previous pregnancy and need a minimum of 2 years to completely restore their nutritional and physical status [31].

Even though this study offers important insight into the effect of birth interval on under-five mortality, the result should be interpreted in light of the following limitations. The matching was done based on the observed variables, there is a possibility of residual confounding. Moreover, DHS is a cross-sectional study and it’s prone to social desirability and recall bias. Despite the abovementioned limitations, the study has the following strengths. First, this study is based on nationally representative DHS data with a high response rate. Secondly, DHS uses a standardized questionnaire for the data collection which is consistent across all 12 countries. Furthermore, this study is the adjustment for potential confounders using the PSM approach in the estimation of the association between birth interval and under-five mortality.

## Conclusion

The short interbirth interval was found significant risk factor for under-five mortality in East African countries. Births to mothers with short birth intervals had a higher chance of dying in the first five years of life. These findings highlight that community and healthcare-based interventions that target enhancing optimal birth spacing should be prioritized and scaled up in East African countries to reduce the burden of under-five mortality in East African countries.

## Data Availability

Data is available online and you can access it from www.measuredhs.com.

## Abbreviations

CI: Confidence Interval
OBI: Optimal Birth Interval
SBI: Short Birth Interval
PSM: Propensity Score Matching
DHS: Demographic and Health Survey
SSA: Sub-Saharan Africa
WHO: World Health Organization
